# Strontium-Cobaltite-Based Perovskite (SrCoO_3_) for Solar-Driven Interfacial Evaporation Systems for Clean Water Generation

**DOI:** 10.3390/nano13081420

**Published:** 2023-04-20

**Authors:** Miao He, Muneerah Alomar, Areej S. Alqarni, Naila Arshad, Muhammad Akbar, Muhammad Yousaf, Muhammad Sultan Irshad, Yuzheng Lu, Qiang Liu

**Affiliations:** 1 Ministry of Education Key Laboratory for the Green Preparation and Application of Functional Materials, Hubei Key Laboratory of Polymer Materials (Hubei University), Collaborative Innovation Center for Advanced Organic Chemical Materials Co-Constructed by the Province and Ministry School of Materials Science and Engineering, Hubei University, Wuhan 430062, China; 2 Department of Physics, College of Sciences, Princess Nourah bint Abdulrahman University, P.O. Box 84428, Riyadh 11671, Saudi Arabia; 3 Collaborative Innovation Centre for Optoelectronic Science & Technology International Collaborative Laboratory of 2D Materials for Optoelectronics Science and Technology of Ministry of Education, Institute of Microscale Optoelectronics, Shenzhen University, Shenzhen 518060, China; nailasehar371@gmail.com; 4 Jiangsu Provincial Key Laboratory of Solar Energy Science and Technology/Energy Storage Joint Research Centre, School of Energy and Environment, Southeast University, Nanjing 210096, China; 5 School of Electronic and Engineering, Nanjing Xiaozhuang University, Nanjing 211171, China

**Keywords:** water scarcity, solar energy, interfacial, evaporation, perovskite, SrCoO_3_, photothermal, fresh water

## Abstract

Solar-driven evaporation technology is often used in areas with limited access to clean water, as it provides a low-cost and sustainable method of water purification. Avoiding salt accumulation is still a substantial challenge for continuous desalination. Here, an efficient solar-driven water harvester that consists of strontium-cobaltite-based perovskite (SrCoO_3_) anchored on nickel foam (SrCoO_3_@NF) is reported. Synced waterways and thermal insulation are provided by a superhydrophilic polyurethane substrate combined with a photothermal layer. The structural photothermal properties of SrCoO_3_ perovskite have been extensively investigated through state-of-the-art experimental investigations. Multiple incident rays are induced inside the diffuse surface, permitting wideband solar absorption (91%) and heat localization (42.01 °C @ 1 sun). Under 1 kW m^−2^ solar intensity, the integrated SrCoO_3_@NF solar evaporator has an outstanding evaporation rate (1.45 kg/m^2^ h) and solar-to-vapor conversion efficiency (86.45% excluding heat losses). In addition, long-term evaporation measurements demonstrate small variance under sea water, illustrating the system’s working capacity for salt rejection (1.3 g NaCl/210 min), which is excellent for an efficient solar-driven evaporation application compared to other carbon-based solar evaporators. According to the findings of this research, this system offers significant potential for producing fresh water devoid of salt accumulation for use in industrial applications.

## 1. Introduction

Fresh water scarcity is emerging as a global concern because of limited resources, an expanding global population, and climate change, which is only expected to worsen, especially in underdeveloped nations and regions [1,2]. Since three quarters of the Earth is covered by oceans, which contain 97% of the total water on the planet, sea water is a seemingly endless resource for clean water [3,4,5]. There is an urgent need to develop an effective, affordable, and long-lasting desalination technology. In recent years, there has been a surge in exploring solar-powered water evaporation owing to its environmentally friendly character [6,7,8]. Unlike bulk-heating-based evaporation, solar-driven interfacial water evaporation can effectively localize thermal energy at the water-air interface, achieving high efficiency of water distillation [9,10,11,12]. To develop efficient solar-powered interfacial water evaporation, cost-effective photothermal materials with high solar absorption and photo-to-thermal conversion are required [11,12]. Several photothermal materials have been reported so far, i.e., metallic nanoparticles, carbon-based materials, wood-based materials, narrow-bandgap semiconductors, and two-dimensional materials [12,13,14,15]. The use of these materials in a variety of two- and three-dimensional evaporators has resulted in a broad spectrum of water evaporation rates under the same solar irradiation (1 kW m^−2^) [16]. Integrating highly efficient photothermal materials for solar energy harvesting and solar steam generation into various two- or three-dimensional evaporators is the focus of one active area of study at present [17].

To date, several photothermal materials have been engineered to accomplish broadband solar absorption that can conform to global conditions [18]. Transition metal oxides are ubiquitous and have unique physical characteristics and vital uses [19]. The perovskite family is a functionally diverse group of compounds having the chemical formula ABO_3_ [8]. Typically, alkali or alkali earth metals and transition metals make up the A and B sites, respectively. However, each compound may contain a variety of atoms on the A or B sites functioning as dopants, increasing functional versatility [20]. SrCoO_3_ is a perovskite oxide and has a perfect perovskite cubic structure with the space group Pm3m, containing alkaline earth metals and transition metals [8]. In SrCoO_3_, conduction band originates from hybridization of the Co 3d and O 2p orbitals, whereas the O 2s and 4p orbitals are positioned below the conduction band. Redox processes involved in oxygen reduction and evolution on SrCoO_3_ are heavily controlled by the electronic structure, and are used for energy storage and conversion applications [21,22,23]. SrCoO_3_ has been investigated for diverse applications, including solar cells [24], sensors [25], electrolyzers [23], and thermochemical water-splitting reactors for H_2_ production [26], due to its high oxygen permeation flux resulting from its high levels of both ionic and electronic conductivities and improved catalytic activity [21,22,23,26]. The non-stoichiometry of oxygen has a major impact on defining its electronic characteristics. Zhao et al. [27] performed in situ ambient pressure X-ray spectroscopy of SrCoO_3_ to reveal its electronic structure and to investigate the electronic structure change with its oxygen stoichiometry and phase, which may then be used to explore the development of SrCoO_3_ for multifunctional applications.

**Figure 1:**
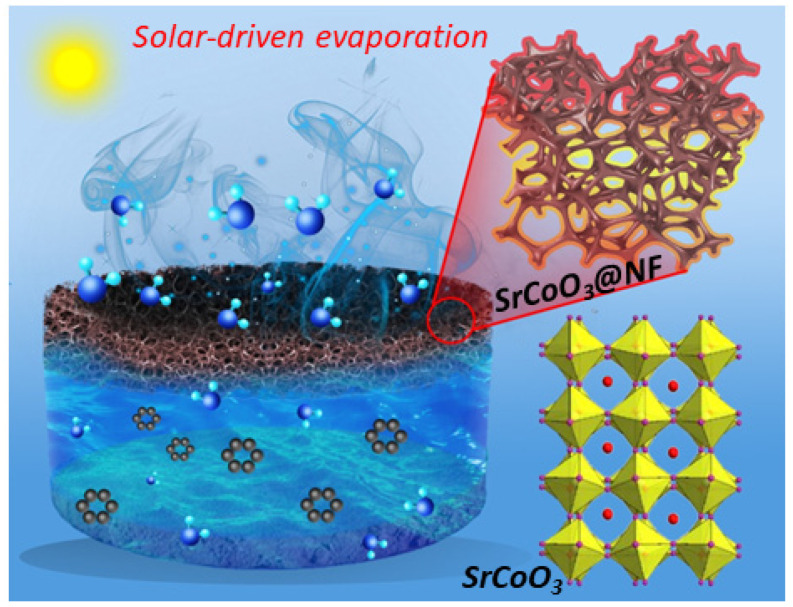
Schematic illustration of strontium-cobaltite-based perovskite (SrCoO_3_) for solar-driven interfacial evaporation systems for clean water generation.

Here, we report an efficient solar-driven water harvester that consists of strontium-cobaltite-based perovskite (SrCoO_3_) anchored on nickel foam (SrCoO_3_@NF) for fresh water generation. The solar evaporator provides synced water transport and good thermal insulation due to a superhydrophilic polyurethane foam (PU) as a substrate combined with a photothermal layer, as shown in Figure 1. The structural photothermal properties of SrCoO_3_ perovskite have been extensively investigated through state-of-the-art experimental investigations. Multiple incident rays are induced inside the diffuse surface, allowing omnidirectional solar absorption (91%) and heat localization (42.01 °C @ 1 sun). Under 1 kW m^−2^ solar intensity, the integrated SrCoO_3_@NF solar evaporator has an outstanding evaporation rate (1.45 kg/m^2^ h) and solar-to-vapor conversion efficiency (86.45% excluding heat losses). In addition, long-term evaporation measurements demonstrate small variance under sea water, illustrating its working capacity for salt rejection (1.3 g NaCl/210 min), which is excellent for an efficient solar-driven evaporation application compared to other carbon-based solar evaporators. This research demonstrates substantial capacity for producing fresh water free of salt ions for industrial uses.

## 2. Materials and Methods

### 2.1. Materials

Cobalt nitrate (Co(NO_3_)_2_·6H_2_O), strontium nitrate (Sr(NO_3_)_2_), and nickel foam substrate (20 cm × 20 cm, sheet) were purchased from Sinopharm Chemical Reagent Co. Ltd., Beijing, China. We purchased polyurethane foam (C_17_H_16_N_2_O_4_) from Changzhou Longisland Automation Technology Co., Ltd., Changzhou, China. All of the compounds maintained a purity level of 99.9% and did not require additional purification. Deionized water was also employed in the experimental procedure.

### 2.2. Synthesis of Strontium-Cobaltite-Based Perovskite (SrCoO_3_)

Cobalt nitrate (Co(NO_3_)_2_6H_2_O), and strontium nitrate (Sr(NO_3_)_2_) were mixed in a solution of ethylenediaminetetraacetic acid (EDTA), ammonium hydroxide (NH_4_OH), and citric acid (C_6_H_8_O_7_). The prepared mixture was heated at 260 °C while being continuously stirred in order to produce a homogeneous gel. The gel was allowed to dry in an oven at 200 °C before being pulverized into a powder. The SrCoO_3_ powder was then exposed to air and heated to 1000 °C for 12 h.

### 2.3. SrCoO_3_-Coated Nickel Foam Solar Evaporator

The fabrication of the self-regenerating perovskite-material-based solar evaporator was achieved using a simple coating method. A specified quantity of the final SrCoO_3_ powder was combined with a specified amount of terpineol (C_10_H_18_O), which is a volatile binder, to form a homogeneous slurry in a mortar pestle. The nickel foam (1.6 mm thickness) was then crafted into a circular shape with a diameter of three centimeters as an interfacial layer for the deposition of SrCoO_3_. A prepared homogeneous slurry of photothermal material was deposited on the nickel foam using a commercial tool. The coated nickel foam was then dried in the oven overnight at 60 °C to remove the terpineol. The nickel foam retained good hydrophilicity and microporous structure after drying. Finally, the SrCoO_3_ anchored on the nickel foam was placed over the PU foam substrate cut to the same diameter as the nickel foam to impart floatability with good water transport and minimum thermal conductivity. Overall, the fabrication was simple, repeatable, readily installable, and self-contained for usage in any location.

### 2.4. Material Characterization Information

Morphological analysis was performed using a field emission scanning electron microscope (FESEM, JSM7100F, JEOL, Tokyo, Japan) to characterize the samples. X-ray diffraction (XRD, Bruker D8 phaser, Coventry, UK) with Cu Kα radiation operating at a current up to 200 mA with a voltage of 40 kV was employed for the phase structural analysis. The elemental compositions were calculated using X-ray photoelectron spectroscopy (XPS) (Thermo Fisher Scientific Escalab 250Xi, Waltham, MA, USA) along with a monochromatic Mg Ka X-ray source. Absorption of the solar spectrum within a spectral range of 250–2500 nm was measured using an ultraviolet-visible near-infrared spectrophotometer (Shimadzu UV-vis-NIR UV-3600 double beam spectrophotometer, Kyoto, Japan) equipped with an integrating sphere. Light absorbance (A) was measured using the A = 1 − transmittance–reflectance formula. Surface temperature data were measured using an infrared camera (FLIR E4 Pro, Deer Park, NY, USA) which included two temperature-sensing thermocouples (K type, hand-held optical meter model). The Fourier-transform infrared (FT-IR) spectrum was obtained using an FT-IR tester (Nicolet iS50, Waltham, MA, USA).

### 2.5. Controlled Solar-Evaporation Experiment

The experimental procedure of vapor generation was performed using a solar simulator (PLS-FX300HU, Beijing Perfect light Technology Co., Ltd., Beijing, China) that can simulate multiple solar intensities up to 6 kW m^−2^. In this experiment, a regular 1.5 G AM spectrum (exactly two standard terrestrial solar spectral irradiance spectra) along with an optical filter was used. The SrCoO_3_@NF solar evaporator was allowed to float freely on the water surface in a beaker (simulated sea water). The entire system was then placed on an advanced electronic balance (Mettler Toledo, ME204, the Strategy, Singapore) with a resolution of 0.001 g, which recorded the time-dependent mass variation to determine the evaporation rate, and exposed under simulated solar radiation (1 kW m^−2^ or one sun). After the stabilization of the evaporation system, the evaporation rates and optimized evaporation (solar to vapor conversion efficiency) were evaluated under one sun illumination (1 kW m^−2^). Inductively coupled plasma-optical emission spectrometry (ICP-OES, PerkinElmer Optima 8000, Waltham, MA, USA) was used to compare the salt concentrations before and after the water was treated. The temperature was maintained at 23 degrees Celsius and relative humidity was maintained at 40 percent throughout the experimental measurements. 

### 2.6. Evaporation Efficiency

Using the following equation [4], we were able to determine the photothermal conversion efficiency (1–2) of the solar-driven evaporation system using SrCoO_3_@NF.

(1) $$\eta_{evap}=\frac{{\dot{m}}_{v}h_{LV}}{q_{solar}}$$

Here, ${\dot{m}}_{v}$ is the evaporation rate (1.45 kg m^−2^ h^−1^) under solar irradiance apart from the evaporation rate of pure water (mass flux), q_solar_ is the incident solar energy (1 kW m^−2^), and $h_{LV}$ is the overall enthalpy of the liquid-to-vapor phase change including sensible heat as well as the phase enthalpy change, which can be measured using the following Equation (2) [4]:

(2) $$h_{LV}=ʎ+C∆T$$

ʎ indicates the latent heat of the phase change while it varies at different temperatures (2430 kJ kg^−1^ K^−1^ at 30 °C, and 2256 kJ kg^−1^ K^−1^ at 100 °C). Water has a specific heat capacity of 4.2 kJ kg^−1^ K^−1^, denoted by C, and its temperature rises gradually, as shown by ΔT. During solar-powered experiments, the humidity was measured at approximately 40% and the temperature was recorded as 23.01 °C. Using the aforementioned formulas, SrCoO_3_@NF can produce vapor at 41.02 °C with a corresponding photothermal conversion efficiency of 86.45% (excluding the evaporation rate; measured in the dark to prevent heat loss due to convection and solar radiation).

## 3. Results & Discussion

### 3.1. Crystal Structure & Compositional Analysis

**Figure 2:**
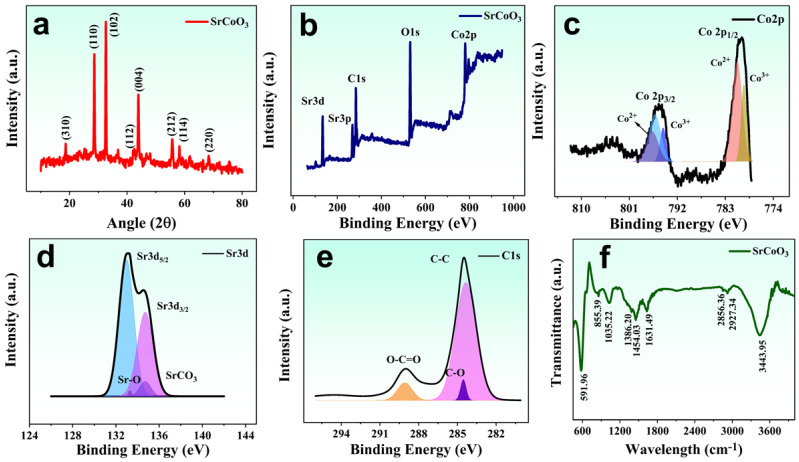
(**a**) XRD spectra of SrCoO_3_. (**b**) XPS survey of SrCoO_3_. High-resolution XPS spectra of (**c**) Co2p, (**d**) Sr3d, and (**e**) C1s. (**f**) FTIR spectrum of perovskite SrCoO_3_.

The efficient and self-regenerating perovskite oxide SrCoO_3_-based solar evaporator concentrates incident-captured light and effectively localizes heat at the interfacial surface, while the lower hydrophilic matrix provides a constant water supply and excellent salt tolerance during the process of continuous evaporation. Crystallographic, elemental, and morphological analysis of the SrCoO_3_ powder was carried out via microscopic techniques, i.e., X-ray diffraction (XRD), X-ray photoelectron spectroscopy (XPS), Fourier-transform infrared spectroscopy (FTIR), and field emission scanning electron microscopy (FESEM). The XRD pattern of the perovskite SrCoO_3_ powder is shown in Figure 2a. All the diffraction peaks are positioned at the Bragg’s angle: 2θ = 18.62°, 28.65°, 32.63°, 44.03, 55.76, 58.27°, and 68.40°, corresponding to the (310), (110), (102), (112), (004), (212), (114), and (220) index planes, respectively, which is in good correspondence with previously reported data [28]. The XRD analysis reveals the formation of the tetragonal crystalline phase of the synthesized SrCoO_3_ powder. Furthermore, X-ray photoelectron spectroscopy was performed to provide insight into the elemental and chemical composition of the SrCoO_3_. Figure 2b shows the full XPS survey of the SrCoO_3,_ showing the existence of several elements, i.e., Co2p, O1s, C1s, Sr3p, and Sr 3d, respectively. The highly resolved XPS spectrum of Co2p is demonstrated in Figure 2c, which has two splits into two distinct peaks due to the two spin-orbit doublets of the cobalt oxides positioned at 780.02 and 795.04 eV, corresponding to the Co 2p (1/2) and Co2p (3/2), respectively. The Co 2p (1/2) spin-orbit doublet is further deconvoluted into two sub-peaks located at 779.54 and 780.69 eV, assigned to the Co^3+^ 2p (1/2) and Co^2+^ 2p (1/2) configurations, respectively, while the Co 2p (3/2) spin-orbit doublet is further split into three distinct peaks at 794.67, 796.08, and 796.80 eV, attributed to the Co^3+^ 2p (3/2) and Co^2+^ 2p (3/2), respectively [8].

A highly resolved spectrum of the Sr 3d core level is shown in Figure 2d, which is split into four sub-peaks at 132.97, 133.32, 134.68, and 134.74 eV. The peaks at 132.97 and 134.68 eV account for the Sr 3d5/2 and Sr 3d3/2 of Sr^2+^, while the other two, at 133.32 and 134.74 eV, correspond to the Sr–O and SrCO_3_ bonds, respectively [8]. The C1s spectrum of SrCoO_3_ shows two main peaks, while three sub-peaks appear at 289.05, 284.32, and 284.53 eV, corresponding to the O–C=O, C–C, and C–O bonds, respectively, as shown in Figure 2e. The presence of various functional groups in SrCoO_3_ was identified by performing Fourier-transform infrared spectroscopy (FTIR). Figure 2f demonstrates the FTIR spectrum of perovskite SrCoO_3_, revealing metal oxide (Sr–O, Co–O) bands with different vibration modes. The Co–O exhibits different vibrational modes ascribed to its different valences, Co^2+^, Co^3+^, and Co^4+^, with bands at the 591.96 cm^−1^ asymmetric vibrational modes of the tetrahedrally coordinated valence (Co^2+^) and octahedrally coordinated valence (Co^3+^). The assigned absorbance bands at 858 cm^−1^ and 1450 cm^−1^ show the existence of the carbonate in the specimen, and correspond to the twisting and vibration, respectively. The band at 855.39 cm^−1^ is due to the twisting and stretching of carbonate, revealing the presence of carbonate in the material. The other bands, 1000 to 3000 cm^−1^, are mainly due to the M–O and M–O–M type vibrational modes (M=Sr, Co) [28]. The band at 1386.20 cm^−1^ corresponds to C–O–C type stretching, while the band at 3443.95 is due to OH stretching.

### 3.2. Surface Morphology

**Figure 3:**
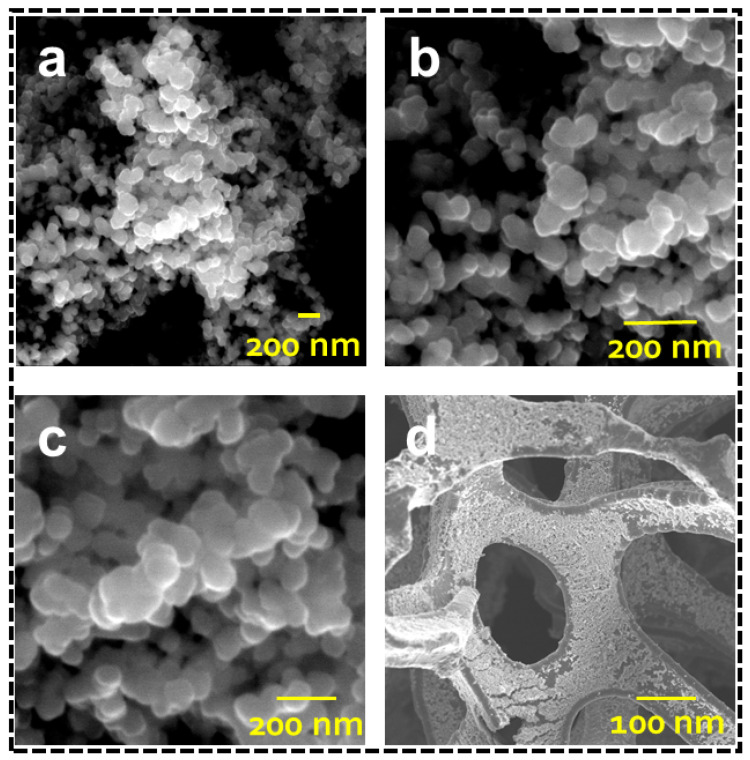
(**a**–**c**) FESEM images of SrCoO_3_ nanoparticles at different resolutions. (**d**) FESEM image of SrCoO_3_-coated nickel foam.

The morphological and microstructural analysis of the prepared perovskite SrCoO_3_ powder and SrCoO_3_ anchored on nickel foam was inspected using field-emission scanning electron microscopy (FESEM). Figure 3a–c shows the FESEM images of the SrCoO_3_, revealing randomly distributed homogeneous shapes with an average size of 100 nm and a high surface-to-volume ratio, which contributes to their enhanced light absorption and improved photothermal conversion. The FESEM image of the SrCoO_3_ anchored on nickel foam (NF) is shown in Figure 3d, showing the highly porous structure of the NF, which facilitates simple water transport and hinders salt resistance. The SrCoO_3_ is uniformly deposited on the walls of the NF, with which imparts a dense texture due to the homogeneously dispersed SrCoO_3_ nanoparticles. This rough, dense morphology facilitates the development of an effective photothermal layer and helps to attain high surface temperatures because of the absorbed solar energy that is converted to heat at the top contact, rather than being allowed to freely diffuse downward into the bulk water.

### 3.3. Solar-Driven Evaporation Efficiency

**Figure 4:**
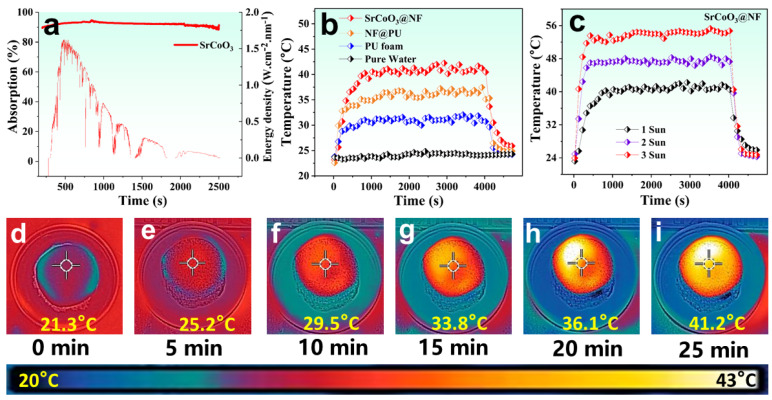
(**a**) UV-Vis absorption spectrum of SrCoO_3_ over wide range (200–2500 nm). (**b**) Surface temperature changes for pure water, PU foam, NF@PU, and SrCoO_3_@NF under 1 kW m^−2^. (**c**) Surface temperature enhancement of SrCoO_3_@NF solar evaporator under different solar intensities. (**d**–**i**) Time-dependent IR images of SrCoO_3_@NF under 1 sun illumination.

Solar-driven interfacial evaporation and water desalination systems have promising potential for optimizing solar energy capture and effective photothermal conversion. Moreover, they support thermal localization at the air-liquid interface, maximized by the black composite surface, which diffuses the incident light inside the photothermal surface. The optical absorption of the SrCoO_3_ was analyzed using UV-Vis spectroscopy in an integrated sphere over the full solar spectrum range (200–2500 nm). The absorption spectrum of the SrCoO_3_ is shown in Figure 4a. The SrCoO_3_ perovskite oxide exhibits enhanced absorption at up to 91% of the entire solar spectrum and reflects just 5%. Its superior absorption assists efficient solar-driven steam generation due to the material’s rough black surface. The potential for solar light capture and the ability to effect photothermal conversion with minimal thermal conduction are crucial to the performance of any system based on solar energy. We created a solar steam generator based on the perovskite SrCoO_3_ material, which has high solar absorption, high solar-to-thermal energy conversion, and low downward thermal conduction. We compared solar evaporation of pure water, PU foam, NF@PU, and SrCoO_3_@NF, for changes in surface temperature under 1 kW m^−2^ for 1 h using thermocouples embedded in the top layers, as shown in Figure 4b.

The enhanced surface temperature of the SrCoO_3_@NF enabled maximum solar harvest, high heat localization inside the top photothermal layer, and outstanding thermal management using hydrophilic PU foam, which allows water transportation only up to the top surface, preventing downward thermal conduction. Therefore, the photothermal layer achieved a peak temperature of 42.21 °C, allowing for a rapid evaporation rate and improved efficiency. This enhanced surface temperature permits the rapid generation of steam, which eventually results in high photothermal conversion efficiency. The surface temperature of SrCoO_3_@NF was measured under multiple sun intensities. At higher solar intensities, the surface temperature of SrCoO_3_@NF is raised, reaching a maximum of 54.2 °C under 3 kW m^−2^ irradiation (Figure 4c). The interfacial thermal accumulation response of SrCoO_3_@NF under 1 kW m^−2^ was also captured using an infrared (IR) camera, as shown in Figure 4d–i. As expected, the SrCoO_3_@NF temperature reached 29.5 °C after 10 min, indicating a fast photothermal response. The creation of thermal localization during evaporation was facilitated by the synergistic impact of the anisotropic low thermal conductivity of SrCoO_3_@NF and the high photothermal conversion of the perovskite material SrCoO_3_. After 25 min of irradiation at a steady 1 kW m^−2^, the SrCoO_3_@NF reached a surface temperature of 41.2 °C. The improved interfacial heat accumulation capability of the SrCoO_3_@NF solar evaporator is responsible for the rapid and dramatic increase in temperature.

**Figure 5:**
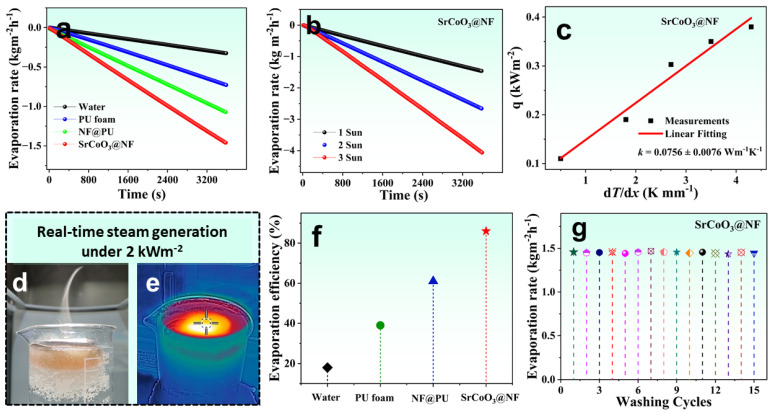
(**a**) Evaporation rate of four solar evaporators under 1 kW m^−2^. (**b**) Evaporation rate profile of SrCoO_3_@NF solar evaporator under multiple solar irradiations. (**c**) Thermal conductivity measurement of SrCoO_3_@NF solar evaporator. (**d**,**e**) Real-time demonstration of vapor generation and respective IR images under 2 sun irradiations of SrCoO_3_@NF solar evaporator. (**f**) Comparative solar-to-vapor conversion efficiencies of four evaporating systems. (**g**) Number of washing cycles vs. evaporation rate of SrCoO_3_@NF solar evaporator.

The rough, dense surface texture of the interfacial photothermal layer of perovskite SrCoO_3_ anchored on NF enables effective thermal localization by inherently dispersing incident solar intensity at the interface, which is the fundamental mechanism of the interfacial solar evaporation structure. We performed a comparative investigation of the four designed systems, i.e., pure water, PU foam, NF@PU, and SrCoO_3_@NF-based solar evaporators, to record evaporation performance under 1 kW m^−2^ solar intensity for 1 h. As expected, high surface temperature enabled fast evaporation and a high evaporation rate. The SrCoO_3_@NF solar evaporator attained a maximum evaporation rate of up to 1.45 kg m^−2^ h^−1^, significantly higher than the other evaporation systems, i.e., pure water (0.32 kg m^−2^ h^−1^), PU foam (0.72 kg m^−2^ h^−1^), and NF@PU (1.08 kg m^−2^ h^−1^), as shown in Figure 5a. The evaporation rate of the self-regenerating SrCoO_3_@NF solar evaporator was also recorded under multiple solar intensities. A maximum evaporation rate of up to 4.06 kg m^−2^ h^−1^ was recorded under 3 k W m^−2^, as shown in Figure 5b. The total efficiency of the solar-powered evaporation system can be optimized by good thermal management of the overall system. In order to design efficient heat accumulation for an effective solar-driven evaporation system, hydrophilic PU foam, which demonstrates high thermal insulation, is used. Here, a thermal conductivity meter (Hot Disk AB, TPS 2500, Sweden) was used to experimentally measure the thermal parameters of the manufactured solar-driven evaporation system. As soon as the power is turned on, a vertical sequence of temperature gradients (dT/dx) is created. The permeation heat transmission rate (q) via the SrCoO_3_@NF system can be found with the use of Fourier Equations (3) and (4) [4], as shown below:

(3) $$q=-k_{1}\frac{dT}{dx}=-k_{1}\frac{T_{2}-T_{1}}{x_{2}-x_{1}}$$

where k_1_ is the thermal conductivity constant (1.05 W m^−1^ K^−1^) [4], x_1_ is the glass slide thickness (3 mm), x_2_ is the SrCoO_3_@NF thickness (30 mm), T_1_ is the temperature of the heating interface, T_2_ is the temperature of the bottom glass of the SrCoO_3_@NF, and T_3_ is the temperature of the top glass of SrCoO_3_@NF.

Given the heat transfer rate and the temperature gradient at thermal equilibrium in the material, the thermal conductivity (k) of SrCoO_3_@NF can be calculated using Equation (4) [4]:

(4) $$k=q\frac{x_{2}}{T_{3}-T_{2}}$$

Figure 5c shows the measured thermal conductivity of SrCoO_3_@NF (0.0756 ± 0.0076 W m^−1^ K^−1^), which is much lower than the thermal conductivity of pure water (0.6 W m^−1^ K^−1^). The high interfacial heat accumulation and superior solar-to-vapor conversion efficiency are the results of optimal solar absorption and minimal thermal conduction. A real-time demonstration of the SrCoO_3_@NF solar evaporator producing vapor under 2 kW m^−2^ intensity is shown in Figure 5d. Figure 5e shows a corresponding infrared picture of a SrCoO_3_@NF solar evaporator operating under 2 sun conditions, revealing a heated interfacial surface with no thermal conduction, which is ideal for a fast evaporation rate in support of effective saltwater desalination. The hydrophilic PU foam effectively limits produced heat, and the infrared picture shows how the interfacial heat buildup is increased over the top matrix and shows effective thermal management compared to existing solar evaporators. The comparative solar-to-vapor conversion efficiency of the pure water, PU foam, NF@PU, and SrCoO_3_@NF were calculated as shown in Figure 5f. The self-regenerating SrCoO_3_@NF had the highest evaporation efficiency, at 86.45%, higher than the pure water (18.22%), PU foam (38.96%), or NF@PU (61.22%). Table 1 represents a comparison with other solar evaporators. Furthermore, the antifouling properties of the SrCoO_3_@NF were assessed by calculating the number of washing cycles as a function of the evaporation rate, as shown in Figure 5g. The SrCoO_3_@NF maintains smooth evaporation rates with little variation, showing excellent scalability for long-term efficacy.

### 3.4. Salt-Rejection Ability

**Figure 6:**
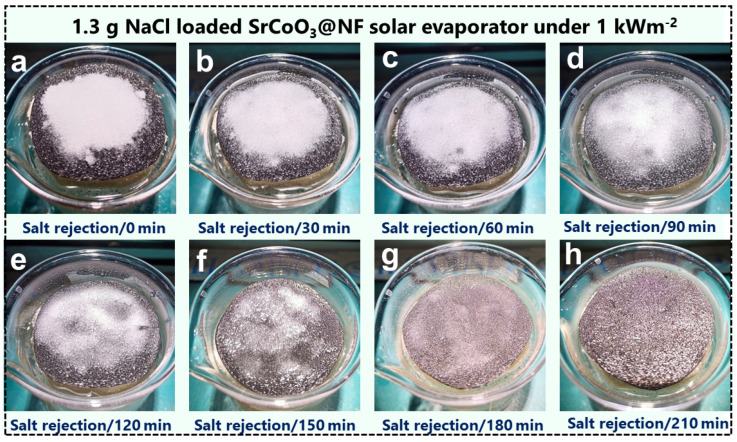
Self-regenerating and salt-resistant performance of SrCoO_3_@NF solar evaporator. (**a**–**h**) Solid NaCl powder (1.3 g) was placed on the top surface of the SrCoO_3_@NF solar evaporator and dissolved in an open porous assembly within 210 min. under 1 kW m^−2^ solar intensity.

The desalination of saltwater uses solar energy, which is a renewable and non-polluting energy source. Nevertheless, during prolonged operation in cooler temperatures, the water channels of evaporation structures tend to get obstructed due to the high salt content of sea water (3.5 wt% NaCl). The optimization of evaporation efficiency is ultimately stifled as a result of salt accumulation. Many effective methods have been proposed to address this problem, such as the introduction of innovative thin evaporation structures at the expense of thermal insulation. These delicate evaporation structures let heat escape from the top interfacial heating layer into the bulk water below. The salt-rejection and self-regeneration ability of the solar-driven SrCoO_3_@NF evaporation structure developed here were investigated through different experimental procedures. The SrCoO_3_@NF solar evaporator was placed in a beaker filled with simulated sea water (3.5 wt% NaCl) and operated for a long period of time at 1 sun solar intensity (1 kW m^−2^) while 1.3 g of solid NaCl was dispersed on the interfacial matrix of the SrCoO_3_@NF solar evaporator, as shown in Figure 6a–h.

In steady-state evaporation conditions, the SrCoO_3_@NF evaporating structure demonstrated exceptional self-regenerating potential by rejecting all of the 1.3 g of solid NaCl in 3.5 h of constant irradiation. The highly porous and interconnected network of the heating and the insulating interface is responsible for the salt rejection and regeneration potential of the designed system. The superwetting feature of microchannels, enhanced by a crosslinking of the interfaces in a centralized macropore arrangement, allows rapid evaporation and simultaneous water transport. Salt ions are transported from a high-concentration to a low-concentration location via diffusion or convection due to the potential gradient caused by the two regions’ varying concentrations. A constant flow of vapor dissolves the solid NaCl, allowing it to migrate through the photothermal layer and into the bulk water below. The SrCoO_3_@NF solar evaporator’s open porous assembly is oriented so that salt does not build up within. Hence, the designed system offers promising potential for industrial-level applicability for sea water desalination.

**Figure 7:**
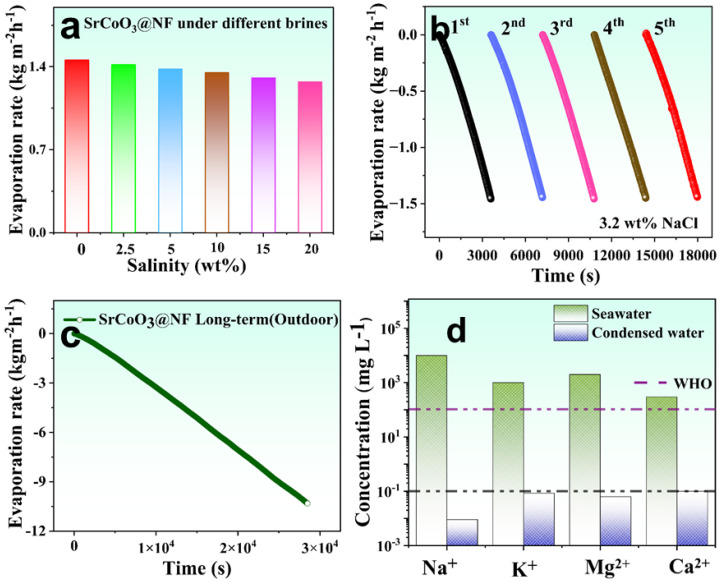
(**a**) Evaporation rates of SrCoO_3_@NF solar evaporator under different salt concentration solutions. (**b**) Cyclic stability of evaporation rate of SrCoO_3_@NF solar evaporator over 5 consecutive cycles. (**c**) Long-term operational stability of SrCoO_3_@NF solar evaporator for 8 h continuously. (**d**) Inductively coupled plasma-optical emission spectrometry (ICP-OES) examination of concentration gradient of primary salt ions in simulated sea water (20 wt%) and condensed water.

The developed SrCoO_3_@NF self-regenerating solar evaporation system shows excellent photothermal conversion, quick water transfer, and fast vapor escape, which offers a potentially useful approach to efficient, effective, and long-term production of fresh water with little impact on the environment. Several brine solutions, including pure water at 2.5, 5, 10, 15, and 20 wt%, were also used to test the SrCoO_3_@NF solar evaporator’s salt-resistance potential without reducing evaporation rates. As shown in Figure 7a, the developed evaporation system performance is not affected by a high salt content, and even higher salt concentrations do not significantly affect the evaporation rate. Furthermore, we investigated the cycle stability of the SrCoO_3_@NF solar evaporator by consistently operating it over 5 cycles under one solar intensity, as shown in Figure 7b. The SrCoO_3_@NF solar evaporator showed no significant change in its evaporation rate. This suggests that the system can be used for a very long time as an efficient solar-driven water evaporation system. However, evaporation structures have several challenges, the most prominent of which is mechanical fragility and structural deformation when operated consistently over a long time period, which has a major impact on the evaporation rate and efficiency of devices. As can be seen in Figure 7c, in order to examine mechanical steadiness and consistency in evaporation efficiency, the SrCoO_3_@NF system was run continuously for 8 h. In simulated saltwater conditions and 1 kW m^−2^ sun intensity, the self-regenerating LSCF@NFP was able to maintain a smooth evaporation rate with little discrepancy, demonstrating great scalability for long-term effectiveness. Additionally, Figure 7d shows the results of inductively coupled plasma-optical emission spectrometry (ICP-OES) measurement of the concentration of four primary salt ions (Na^+^, K^+^, Ca^2+^, Mg^2+^) in stimulated sea water and condensed water, which was performed to evaluate the purifying potential of the developed system. After desalination, the concentration of salt ions drops by three to four orders of magnitude, far below the standards for drinking water of the World Health Organization (WHO) and the US Environmental Protection Agency (EPA). Hence, the SrCoO_3_@NF solar evaporator optimally produces potable fresh water, expanding its potential industrial applications.

## 4. Conclusions

In summary, we successfully synthesized strontium-cobaltite-based perovskite (SrCoO_3_) nanoparticles via the sol-gel technique. A detailed structural investigation was performed to fabricate a solar-driven water harvester composed of strontium-cobaltite-based perovskite (SrCoO_3_) anchored on nickel foam (SrCoO_3_@NF). Synced water transport and good thermal insulation are provided by a superhydrophilic polyurethane substrate combined with a photothermal layer. Multiple incident rays are induced inside the diffuse surface, allowing omnidirectional solar absorption (91%) and heat localization (42.01 °C @ 1 sun). Under 1 kW m^2^ solar intensity, the integrated SrCoO_3_@NF solar evaporator has an outstanding evaporation rate (1.45 kg/m^2^ h) and solar-to-vapor conversion efficiency (86.45% excluding heat losses). In addition, long-term evaporation measurements demonstrate low variance under sea water, illustrating the system’s working capacity for salt rejection (1.3 g NaCl/210 min). This research demonstrates a substantial capacity to produce fresh water free of salt ions for industrial uses.

## Figures and Tables

**Table 1:** Comparison of solar-driven evaporation performance of SrCoO_3_@NF with other solar evaporators.

Sr. No	Solar Driven System	Evaporation Rate (Kg m^−2^ h^−1^)	Efficiency (%)	Ref.
1.	Carbon-black-coated polymethylmethacrylate (PMMA) layer, (CB/PMMA)	1.3	72	[29]
2.	Fe_3_O_4_-coated delignified wood (Fe-D-Wood)	1.3	73	[30]
3.	Graphene oxide/cellulose ester membrane (GO/MCE)	1.3	86	[31]
4.	Carbonized pencil waste evaporator	1.2	82.2	[32]
5.	In situ alkalized 3D carbon foam (CF)	1.26	80.1	[33]
6.	Black sand	1.43	81	[34]

## Data Availability

The data request will be furnished upon reasonable request.

## References

[1] Irshad M.S., Arshad N., Wang X. (2020). Nanoenabled Photothermal Materials for Clean Water Production. Glob. Chall..

[2] Arshad N., Ahmed I., Irshad M.S., Li H.R., Wang X., Ahmad S., Sharaf M., Firdausi M., Zaindin M., Atif M. (2020). Super Hydrophilic Activated Carbon Decorated Nanopolymer Foam for Scalable, Energy Efficient Photothermal Steam Generation, as an Effective Desalination System. Nanomaterials.

[3] Song C., Irshad M.S., Jin Y., Hu J., Liu W. (2022). Arabic-Dome-Inspired Hierarchical Design for Stable and High-Efficiency Solar-Driven Seawater Desalination. Desalination.

[4] Dao V.-D., Vu N.H., Yun S. (2020). Recent Advances and Challenges for Solar-Driven Water Evaporation System toward Applications. Nano Energy.

[5] Irshad M.S., Hao Y., Arshad N., Alomar M., Lin L., Li X., Wageh S., Al-Hartomy O.A., Al-Sehemi A.G., Dao V.-D. (2023). Highly Charged Solar Evaporator toward Sustainable Energy Transition for In-Situ Freshwater & Power Generation. Chem. Eng. J..

[6] Guo Z., Zhou W., Arshad N., Zhang Z., Yan D., Irshad M.S., Yu L., Wang X. (2022). Excellent Energy Capture of Hierarchical MoS2 Nanosheets Coupled with MXene for Efficient Solar Evaporators and Thermal Packs. Carbon N. Y..

[7] Irshad M.S., Arshad N., Wang X., Li H.R., Javed M.Q., Xu Y., Alshahrani L.A., Mei T., Li J. (2021). Intensifying Solar Interfacial Heat Accumulation for Clean Water Generation Excluding Heavy Metal Ions and Oil Emulsions. Sol. RRL.

[8] Irshad M.S., Arshad N., Zhang J., Song C., Mushtaq N., Alomar M., Shamim T., Dao V.-D., Wang H., Wang X. (2023). Wormlike Perovskite Oxide Coupled with Phase-Change Material for All-Weather Solar Evaporation and Thermal Storage Applications. Adv. Energy Sustain. Res..

[9] Yu F., Guo Z., Xu Y., Chen Z., Irshad M.S., Qian J., Mei T., Wang X. (2020). Biomass-Derived Bilayer Solar Evaporator with Enhanced Energy Utilization for High-Efficiency Water Generation. ACS Appl. Mater. Interfaces.

[10] Liu G., Yu F., Irshad M.S., Xiong X., Guo Z., Wang J., Xiao B., Lin L., Wang X. (2022). Biomass-Inspired Solar Evaporator for Simultaneous Steam and Power Generation Enhanced by Thermal-Electric Effect. Energy Technol..

[11] Wei Z., Arshad N., Irshad M.S., Idrees M., Ahmed I., Li H., Qazi H.H., Yousaf M., Alshahrani L.A., Lu Y. (2021). A Scalable Prototype by In Situ Polymerization of Biodegradables, Cross-Linked Molecular Mode of Vapor Transport, and Metal Ion Rejection for Solar-Driven Seawater Desalination. Crystals.

[12] Wei Z., Arshad N., Hui C., Irshad M.S., Mushtaq N., Hussain S., Shah M., Naqvi S.Z.H., Rizwan M., Shahzad N. (2022). Interfacial Photothermal Heat Accumulation for Simultaneous Salt Rejection and Freshwater Generation; an Efficient Solar Energy Harvester. Nanomaterials.

[13] Irshad M.S., Wang X., Abbasi M.S., Arshad N., Chen Z., Guo Z., Yu L., Qian J., You J., Mei T. (2021). Semiconductive, Flexible MnO_2_ NWs/Chitosan Hydrogels for Efficient Solar Steam Generation. ACS Sustain. Chem. Eng..

[14] Yu F., Chen Z., Guo Z., Irshad M.S., Yu L., Qian J., Mei T., Wang X. (2020). Molybdenum Carbide/Carbon-Based Chitosan Hydrogel as an Effective Solar Water Evaporation Accelerator. ACS Sustain. Chem. Eng..

[15] Wang J., Shamim T., Arshad N., Irshad M.S., Mushtaq M.N., Zhang C., Yousaf M., Alshahrani L.A., Akbar M., Lu Y. (2022). In Situ Polymerized Fe_2_O_3_@ PPy/Chitosan Hydrogels as a Hydratable Skeleton for Solar-Driven Evaporation. J. Am. Ceram. Soc..

[16] Tao P., Ni G., Song C., Shang W., Wu J., Zhu J., Chen G., Deng T. (2018). Solar-Driven Interfacial Evaporation. Nat. Energy.

[17] Zhu Y., Tian G., Liu Y., Li H., Zhang P., Zhan L., Gao R., Huang C. (2021). Low-Cost, Unsinkable, and Highly Efficient Solar Evaporators Based on Coating MWCNTs on Nonwovens with Unidirectional Water-Transfer. Adv. Sci..

[18] Wu S.-L., Chen H., Wang H.-L., Chen X., Yang H.-C., Darling S.B. (2021). Solar-Driven Evaporators for Water Treatment: Challenges and Opportunities. Environ. Sci. Water Res. Technol..

[19] Guo X.-L., Ding Z.-Y., Deng S.-M., Wen C.-C., Shen X.-C., Jiang B.-P., Liang H. (2018). A Novel Strategy of Transition-Metal Doping to Engineer Absorption of Carbon Dots for near-Infrared Photothermal/Photodynamic Therapies. Carbon N. Y..

[20] Shangguan Z., Zheng X., Zhang J., Lin W., Guo W., Li C., Wu T., Lin Y., Chen Z. (2020). The Stability of Metal Halide Perovskite Nanocrystals—A Key Issue for the Application on Quantum-Dot-Based Micro Light-Emitting Diodes Display. Nanomaterials.

[21] Tahini H.A., Tan X., Zhou W., Zhu Z., Schwingenschlögl U., Smith S.C. (2017). Sc and Nb Dopants in SrCoO_3_ Modulate Electronic and Vacancy Structures for Improved Water Splitting and SOFC Cathodes. Energy Storage Mater..

[22] Aguadero A., Pérez-Coll D., Alonso J.A., Skinner S.J., Kilner J. (2012). A New Family of Mo-Doped SrCoO_3_−δ Perovskites for Application in Reversible Solid State Electrochemical Cells. Chem. Mater..

[23] Duan D., Fang X., Li K. (2022). A Peroxidase-like Nanoenzyme Based on Strontium(II)-Ion-Exchanged Prussian Blue Analogue Derivative SrCoO_3_/Co_3_O_4_ Nanospheres and Carbon Quantum Dots for the Colorimetric Detection of Tigecycline in River Water. Talanta.

[24] Xu M., Wang W., Liu Y., Zhong Y., Xu X., Sun Y., Wang J., Zhou W., Shao Z. (2019). An Intrinsically Conductive Phosphorus-Doped Perovskite Oxide as a New Cathode for High-Performance Dye-Sensitized Solar Cells by Providing Internal Conducting Pathways. Sol. RRL.

[25] He J., Xu X., Sun H., Miao T., Li M., Zhou S., Zhou W. (2023). Participation of Lattice Oxygen in Perovskite Oxide as a Highly Sensitive Sensor for P-Phenylenediamine Detection. Molecules.

[26] Cavallaro A., Wilson G.E., Kerherve G., Cali E., van den Bosch C.A.M., Boldrin P., Payne D., Skinner S.J., Aguadero A. (2021). Analysis of H_2_O-Induced Surface Degradation in SrCoO_3_-Derivatives and Its Impact on Redox Kinetics. J. Mater. Chem. A.

[27] Zhao J., Luo Y., Wang J.-O., Qian H., Liu C., He X., Zhang Q., Huang H., Zhang B., Li S. (2019). Electronic Structure Evolutions Driven by Oxygen Vacancy in SrCoO_3−*x*_ Films. Sci. China Mater..

[28] Verma A.K., Mahato D.K. (2022). Structural and FTIR Analysis of SrCoO_3_ Perovskite Ceramics. IOSR J. Appl. Phys..

[29] Xu W., Hu X., Zhuang S., Wang Y., Li X., Zhou L., Zhu S., Zhu J. (2018). Flexible and Salt Resistant Janus Absorbers by Electrospinning for Stable and Efficient Solar Desalination. Adv. Energy Mater..

[30] Song L., Zhang X.-F., Wang Z., Zheng T., Yao J. (2021). Fe_3_O_4_/Polyvinyl Alcohol Decorated Delignified Wood Evaporator for Continuous Solar Steam Generation. Desalination.

[31] Hou B., Kong D., Chen Z., Shi Z., Cheng H., dong Guo D., Wang X. (2019). Flexible Graphene Oxide/Mixed Cellulose Ester Films for Electricity Generation and Solar Desalination. Appl. Therm. Eng..

[32] Lu Y., Dai T., Fan D., Min H., Ding S., Yang X. (2020). Turning Trash into Treasure: Pencil Waste–Derived Materials for Solar-Powered Water Evaporation. Energy Technol..

[33] Qiu P., Liu F., Xu C., Chen H., Jiang F., Li Y., Guo Z. (2019). Porous Three-Dimensional Carbon Foams with Interconnected Microchannels for High-Efficiency Solar-to-Vapor Conversion and Desalination. J. Mater. Chem. A.

[34] Zhang Q., Xiao X., Wang G., Ming X., Liu X., Wang H., Yang H., Xu W., Wang X. (2018). Silk-Based Systems for Highly Efficient Photothermal Conversion under One Sun: Portability, Flexibility, and Durability. J. Mater. Chem. A.

